# The Role of Adverse Childhood Experiences in Severe Mental Illness and Criminal Behavior

**DOI:** 10.1192/j.eurpsy.2026.10953

**Published:** 2026-07-31

**Authors:** C. O. Areias, J. F. Silva, R. M. Pinhel, A. I. Machado

**Affiliations:** ULS Santo António, Porto, Portugal

## Abstract

**Introduction:**

Adverse Childhood Experiences (ACE) are traumatic events that occur during childhood and have the potential to negatively impact an individual’s mental and physical health throughout their life, and may also elevate the risk of future involvement in criminal activity. The Family ACE Questionnaire is a self-report tool designed to assess 10 types of adverse experiences in childhood: physical, emotional, or sexual abuse; emotional or physical neglect; exposure to domestic violence; substance abuse in the family environment; parental divorce or separation; incarceration of a family member; and mental illness or suicide.

**Objectives:**

This study aims to compare the prevalence of ACE among patients admitted to acute psychiatric inpatient units and those admitted to forensic psychiatric services. Additionally, it seeks to explore the relationship between ACE exposure and the development of psychoactive substance use, comorbid medical conditions, and criminal behavior.

**Methods:**

A cross-sectional study is being conducted using data collected from the anonymous and confidential self-report of the validated Portuguese version of the shortened ACE questionnaire. The sample consists of male inpatients aged 65 years or younger, admitted to either the acute psychiatric or forensic psychiatric units of Hospital Magalhães Lemos. In the forensic psychiatry unit, data is being collected between August 2024 and December 2025, as part of the standard admission protocol. In the acute unit, data is being collected between July and December 2025. In addition to the questionnaire responses, data are also collected on age, psychiatric diagnosis, comorbid medical conditions, psychoactive substance use, criminal history, and age at first offense. Statistical analysis will be conducted using SPSS.

**Results:**

A total sample of 100 patients is anticipated, comprising 50 from the forensic psychiatry unit and 50 from the acute psychiatric unit. As data collection is still ongoing, statistical analysis remains in progress.

**Conclusions:**

A high prevalence of ACE is expected in both groups, with a higher prevalence anticipated among forensic psychiatric inpatients. This may indicate an association between elevated ACE scores and a greater likelihood of criminal behavior, regardless of the presence of mental illness. Associations are also expected between higher ACE scores and substance use, as well as with medical conditions, particularly cardiovascular diseases. Incorporating the impact of ACE is important when developing strategies to enhance mental health among adults, and potentially in the prevention of criminal behavior among individuals with severe mental illness.

**Disclosure of Interest:**

None Declared

